# Supplementary material for: Identification of photoperiod-induced specific miRNAs in the adrenal glands of Sunite sheep (Ovis aries)
Source: Front Vet Sci. 2022 Jul 22;9:888207. doi: 10.3389/fvets.2022.888207 (PMC9354845; doi:10.3389/fvets.2022.888207)
Supplement: Supplementary file 1 [file Data_Sheet_1.ZIP › Supplementary file/miRDeep2 file.zip!/miRDeep2 file/Novel_Structure/SP42A2/Novel_311.pdf]

Provisional ID	:	NC_019459.2_6461
Score total	:	1.9
Score for star read(s)	:	-1.3
Score for read counts	:	0
Score for mfe	:	1.6
Score for randfold	:	1.6
Score for cons. seed	:	
Total read count	:	12
Mature read count	:	12
Loop read count	:	0
Star read count	:	0

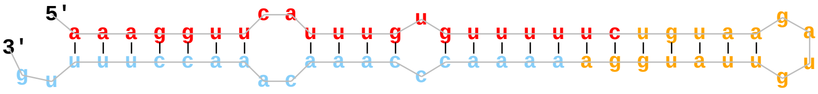

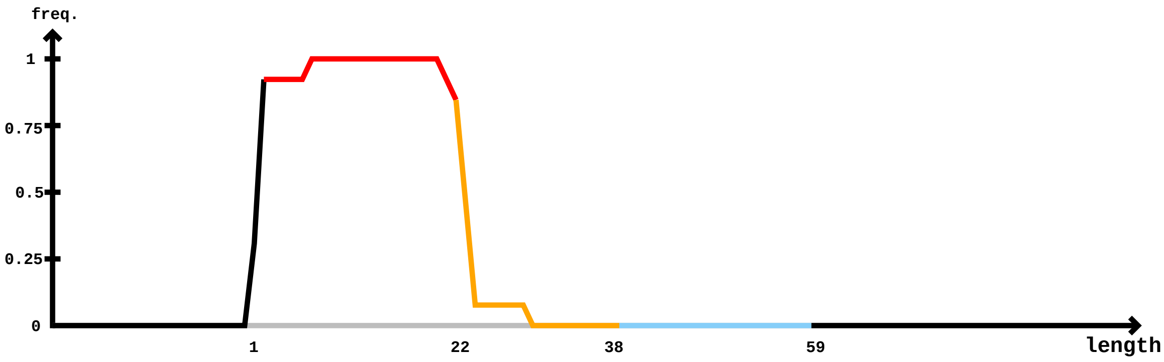


```
                    Mature                              Star
5'- uggaaauacuagauuggccaaaagguucauuuguguuuuucuguaagauguuauggaaaaacccaaacaaaccuuuuggccaacccaauauaaaacuacucuguauaaguc   -3'    exp
    .....((((.(((((((((((((((((..((((.((((((((((((....)))))))))))).))))..))))))))))))))................)))))))).....     reads      mm        sample
    ...................aaaagguucauuuguguuuu........................................................................     1          0         seq
    ...................aaaagguucauuugGguuuuu.......................................................................     1          1         seq
    ...................aaaagguucauuuguguuuuuc......................................................................     2          0         seq
    ....................aaagguucauuuguguuuuuc......................................................................     7          0         seq
    ....................aaagguucauuugAguuuuucu.....................................................................     1          1         seq
    .........................uucauuugAguuuuucuguaaga...............................................................     1          1         seq
```
